# On the kinetics of anaerobic power

**DOI:** 10.1186/1742-4682-9-29

**Published:** 2012-07-25

**Authors:** John F Moxnes, Kjell Hausken, Øyvind Sandbakk

**Affiliations:** 1 Department for Protection, Norwegian Defence Research Establishment, P.O. Box 25, 2007, Kjeller, Norway; 2 Faculty of Social Sciences, University of Stavanger, 4036, Stavanger, Norway; 3 Department of Human Movement Science, Norwegian University of Science and Technology, 7491, Trondheim, Norway

**Keywords:** Blood lactate, Cross-country skier, Mathematical model, Oxygen uptake, Phosphocreatine

## Abstract

**Background:**

This study investigated two different mathematical models for the kinetics of anaerobic power. Model 1 assumes that the work power is linear with the work rate, while Model 2 assumes a linear relationship between the alactic anaerobic power and the rate of change of the aerobic power. In order to test these models, a cross country skier ran with poles on a treadmill at different exercise intensities. The aerobic power, based on the measured oxygen uptake, was used as input to the models, whereas the simulated blood lactate concentration was compared with experimental results. Thereafter, the metabolic rate from phosphocreatine break down was calculated theoretically. Finally, the models were used to compare phosphocreatine break down during continuous and interval exercises.

**Results:**

Good similarity was found between experimental and simulated blood lactate concentration during steady state exercise intensities. The measured blood lactate concentrations were lower than simulated for intensities above the lactate threshold, but higher than simulated during recovery after high intensity exercise when the simulated lactate concentration was averaged over the whole lactate space. This fit was improved when the simulated lactate concentration was separated into two compartments; muscles + internal organs and blood. Model 2 gave a better behavior of alactic energy than Model 1 when compared against invasive measurements presented in the literature. During continuous exercise, Model 2 showed that the alactic energy storage decreased with time, whereas Model 1 showed a minimum value when steady state aerobic conditions were achieved. During interval exercise the two models showed similar patterns of alactic energy.

**Conclusions:**

The current study provides useful insight on the kinetics of anaerobic power. Overall, our data indicate that blood lactate levels can be accurately modeled during steady state, and suggests a linear relationship between the alactic anaerobic power and the rate of change of the aerobic power.

## Background

During human exercise, it is well known that intracellular adenosine triphosphate (ATP) can be produced aerobically in the mitochondria by oxidative phosphorylation, anaerobically due to glycolysis or glycogenolysis, or by the breakdown of phosphocreatine (PCr) into Creatine (Cr) in the Creatine Kinease (CK) reaction. With the aerobic energy as input, the current paper develops mathematical models that simulate the kinetics of anaerobic ATP production and power.

The rate of oxygen (O_2_) consumption can be set to the sum of a constant rate (resting rate of O_2_ consumption), a rate due to unloaded body movements and at a rate that is used to perform work [1]. For moderate constant work rates, the O_2_ consumption increases to a steady state condition. However, at the onset of exercise or due to a change in work rate, there is a rate of change of VO_2_ (or aerobic power) that is named as the VO_2_ kinetics. Pulmonary rate of O_2_ consumption (VO_2p_) has been used as a proxy for VO_2_. For moderate intensity exercise at constant work rate below the lactate threshold three distinct phases have been observed for VO_2p_. Phase I is the cardio dynamic phase, which represents the circulatory transit delay from muscles to lungs. Phase II is the mono exponential increase of VO_2p_. This phase reflects the adjustment of VO_2_ due to the use of active skeletal muscles. Phase III is the steady state phase of VO_2p_ and VO_2_ during moderate exercise intensities [2,3]. For work rates associated with sustained acidosis, the mono-exponential component is slowed compared with lower intensities below the lactate threshold. In addition, a delayed slow component is superimposed. The slow component begins around 100-200 s into the exercise and can result in either a delayed sub-maximal steady state or a steady state equal to the maximal oxygen uptake (VO_2_ max) [4]. However, the mechanism of this slow component has not been resolved [5]. In this paper, we model phase II and III because only these two phases are considered relevant for the anaerobic alactic power model.

When exercise intensity increases and the rate of ATP production by oxidative sources becomes insufficient, anaerobic ATP production is required. When ATP is produced by glycolysis or glycogenolysis the endpoint is pyruvate, which can be reduced to lactate or oxidized to CO_2_ or H_2_O. The blood lactate concentration in the lactate pool is the result of the appearance of lactate from working muscles and various tissues and the disappearance of lactate in the skeletal muscles, the heart, the liver and the kidney cortex [6-9]. During steady state, lactate production (influx) is equal to lactate removal (outflux). As a result, the lactate concentration in the lactate pool stays constant, and the rate of oxygen consumption is the measure of the whole body energy expenditure regardless of the magnitude of lactate production and removal or the absolute blood lactate concentration [10]. The concept of a maximal lactate steady state can be defined as the highest level of intensity where a steady state condition of lactate can be obtained, which is also referred to as the lactate threshold. At exercise intensities above the lactate threshold the rise in the lactate concentration could be attributed to an increase in the rate of lactate appearance or the result of a decrease in the rate of lactate disappearance [10].

The maximum anaerobic energy that can be utilized is proportional to the sum of Cr and lactate that can accumulate in the body. PCr is an energy buffer that supports the transient failure of other metabolic pathways to support ATP production. The equilibrium constant of the CK reaction is around 20 and the slightest drop in ATP allows the reaction to proceed to ATP production [11]. Thus, the ATP concentration stays nearly constant until almost all the PCr is utilized. Rossiter et al. [12] found that the PCr levels follow an exponential time course after changes in work rate before approaching a steady state condition at moderate exercise intensities. In such cases, a strong similarity has been reported for the time constants of the VO_2_ kinetics and the PCr consumption [12]. Margaria [13] was the first to propose a hydraulic model for examining the whole body energy process during exercise. Despite this breakthrough, Margaria’s model was not quantified. Morton [14] presented a generalization of this model that was solved mathematically and compared with experimental data. Here Morton [14] modeled aerobic power and alactic anaerobic power with an exponential time devolvement without any anaerobic lactic power during steady state exercises below the lactate threshold. However, for exercise intensities that are above the lactate threshold, the anaerobic glycolytic energy supply is significant. The association between PCr and VO_2_ rate constants for exercises at such intensities has not yet been systematically reported. Furthermore, during recovery after high intensity exercise, the level of PCr must be restored, the pH must be re-established and ADP removed. While the PCr recovery is mainly due to oxidative ATP synthesis, the PCr stores may be rebuilt by anaerobic glycolysis [15-17]. Altogether, these insights provide a theoretical background for the models developed in this paper.

The O_2_-deficit formula of Medbø et al. [18] is an alternative model for the anaerobic power that accounts for lactic and alactic anaerobic power in which the chemical coupling efficiencies are assumed to be similar. Medbø et al. [18] suggested that the O_2_-deficit can be calculated by assuming that the metabolic power at intensities above VO_2_max can be estimated by extrapolating the steady state linear relationship between work rate and VO_2_ at submaximal intensities. The validity of the O_2_-deficit method has been widely debated [e.g., [19-21]. However, a rationale for the O_2_-deficit model is the assumption that the chemical coupling efficiencies of the three sources of ATP synthesis are similar.

The current study investigated two different mathematical models for the kinetics of anaerobic power during whole body exercise at different intensities. In order to test the models during exercise, oxygen uptake and blood lactate concentration were measured while a cross country skier ran with poles on a treadmill. Aerobic power, based on oxygen uptake measurements, was used as input. The lactic anaerobic power was calculated with a model presented by Moxnes and Hausken [10] using lactate concentration averaged over the whole lactate space, and with a model of Moxnes and Sandbakk [22] where the lactate concentration was separated into two compartments: muscles + internal organs and blood. The current study has input from these two previous studies, and focuses on alactic power as a novel contribution. Therefore, the power due to PCr break down was calculated theoretically and compared against the results of Jeneson et al. [23]. Finally, the models were used to compare PCr break down during continuous and interval exercise.

## Methods

### Overall design

The current study simulated the kinetics of anaerobic powers during whole body exercise at different exercise intensities through the use of Mathematica 8 (Wolfram Research Inc., Champaign, IL, USA). In a first model (Model 1), we suggested that for all exercise intensities, the work power would be linear to the work rate. As an alternative model (Model 2) where the lactic power and energy were the same as for Model 1, we hypothesized that the alactic anaerobic power would be proportional to the rate of change of the aerobic power [24,25]. For both models we used a first order differential equation for the time development of aerobic power as a function of work rate. As most parameters in the models needed to be fitted to each individual athlete, we fitted all parameters to a male Norwegian national level cross-country skier, with a body mass of *m* = 77.5 kg, body height of 181 cm and 600 h of endurance training per year. Thereafter, the simulations were compared with experimental data where this skier was running with poles on a treadmill (see details below).

### The aerobic and anaerobic powers

ATP is produced by three different sources:

(1a) $$\underset{\begin{array}{l} metabolic\\ power \end{array}}{\underset{}{Q\left(t\right)}}-\underset{\begin{array}{l} resting\\ power \end{array}}{\underset{}{Q_{r}}}\overset{model}{=}\underset{\begin{array}{l} \;aerobic\;\\ power \end{array}}{\underset{}{Q_{a}^{w}\left(t\right)}}+\underset{\begin{array}{l} power\;from\;\\ G \end{array}}{\underset{}{Q_{G}\left(t\right)}}+\underset{\begin{array}{l} power\;from\\ CK \end{array}}{\underset{︸}{Q_{\mathrm{CK}}\left(t\right)}},\left(a\right)$$

(1b) $$\underset{\begin{array}{l} rate\;of\;ATP\\ consumed \end{array}}{\underset{}{I\left(t\right)}}-\underset{\begin{array}{l} resting\\ rate \end{array}}{\underset{}{I_{r}}}\overset{model}{=}\underset{\begin{array}{l} \;rate\;of\;ATP\;\\ consumed\\ with\;ATP\;\\ produced\;\\ aerobically \end{array}}{\underset{}{I_{a}^{w}\left(t\right)}}+\underset{\begin{array}{l} rate\;of\;ATP\\ \;consumed\;\\ with\;ATP\\ produced\;from\\ G \end{array}}{\underset{}{I_{G}\left(t\right)}}+\underset{\begin{array}{l} rate\;of\;ATP\;\\ consumed\;\\ with\;ATP\\ produced\;from\\ CK \end{array}}{\underset{︸}{I_{\mathrm{CK}}\left(t\right)}}$$

In these equations, “model” means model assumption. *Q*_*r*_ is the resting metabolic power, set to 80 J/s based on oxygen uptake measurements of this skier during rest in our laboratory. $Q_{G}$ is ATP production by glycolysis/glycogenolysis and CK by phosphocreatine break down to creatine. $Q_{a}^{w}$ is aerobic power due to internal and external work. The aerobic power is $Q_{a}=Q_{r}+Q_{a}^{w}$.

For each ATP used (produced and consumed) heat and work are outputs. Heat when producing ATP differs between aerobic and anaerobic sources. However, heat when consuming ATP is the same for the aerobic and anaerobic sources. We defined work power and heat power due to oxidative phosphorylation (a), glycolysis/glycogenolysis (G) and phosphocreatine break down to creatine (CK) as:

(2a) $$\underset{\begin{array}{l} Work\\ power \end{array}}{\underset{}{P_{a}}}\overset{def}{=}\eta \eta_{a}Q_{a}^{w},\underset{\begin{array}{l} Heat\\ power \end{array}}{\underset{}{H_{a}^{w}}}=Q_{a}^{w}-P_{a}=Q_{a}^{w}\left(1-\eta \eta_{a}\right)$$

(2b) $$\underset{\begin{array}{l} Work\\ power \end{array}}{\underset{}{P_{G}}}\overset{def}{=}\eta \eta_{G}Q_{G},\underset{\begin{array}{l} Heat\\ power \end{array}}{\underset{}{H_{G}}}=Q_{G}-P_{G}=Q_{G}\left(1-\eta \eta_{G}\right)$$

(2c) $$\underset{\begin{array}{l} Work\\ power \end{array}}{\underset{}{P_{\mathrm{CK}}}}\overset{def}{=}\eta \eta_{\mathrm{CK}}Q_{\mathrm{CK}},\underset{\begin{array}{l} Heat\\ power \end{array}}{\underset{}{H_{\mathrm{CK}}}}=Q_{\mathrm{CK}}-P_{\mathrm{CK}}=Q_{\mathrm{CK}}\left(1-\eta \eta_{\mathrm{CK}}\right)$$

(2d) $$\underset{\begin{array}{l} Total\\ work\;power \end{array}}{\underset{}{P_{T}}}\overset{def}{=}P_{a}+P_{G}+P_{\mathrm{CK}}=\eta \eta_{a}Q_{a}^{w}+\eta \eta_{G}Q_{G}+\eta \eta_{\mathrm{CK}}Q_{\mathrm{CK}}\text{,}$$

(2e) $$\underset{\begin{array}{l} Heat\;power\\ due\;to\;work \end{array}}{\underset{}{H}}\overset{def}{=}H_{a}^{w}+H_{G}+H_{\mathrm{CK}}$$

where “def” means definition. *η*_*a*_*, η*_*G*_ and *η*_*CK*_ are the chemical coupling efficiencies when producing ATP aerobically, anaerobically by glycolysis/glycogenolysis (G), and anaerobically by CK, respectively. The two chemical coupling efficiencies *η*_*G*_ and *η*_*a*_ are similar, whereas *η*_*CK*_ is larger [26]. *η* is the mechanical efficiency, which means the work per unit use of energy of 1 ATP. *P*_*T*_ includes both internal and external work.

**Figure 1:**
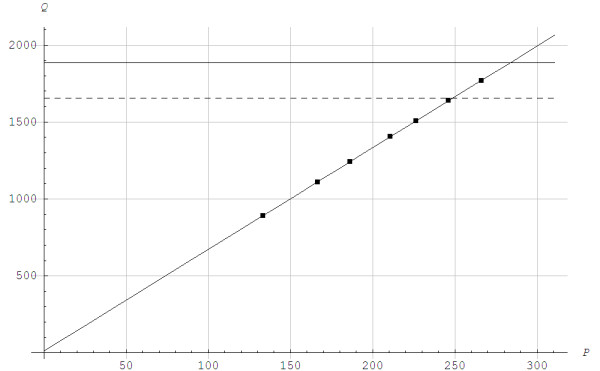
**The steady state aerobic power**${\bar{Q}}_{a}$**and the virtual steady state**$\bar{\tilde{Q}}$**in J/s as functions of work rate *P* in J/s when running with poles at the angle of inclination of 0.105 radians for 5-min steady state work rates.** The lower horizontal line is the lactate threshold = 1654 J/s, and the upper horizontal line the maximal aerobic power $Q_{\max}=1886J/s$. ■: Experimental data, $v_{1}=2.08\;\text{m}/\text{s},{\bar{Q}}_{\mathrm{vir}}=0.59\;Q_{\max}$, $v_{2}=2.33\;\text{m}/\text{s},{\bar{Q}}_{\mathrm{vir}}=0.66\;Q_{\max}$, $v_{3}=2.64\;\text{m}/\text{s},{\bar{Q}}_{\mathrm{vir}}=0.74Q_{\max}$, $v_{4}=2.83\;\text{m}/\text{s},{\bar{Q}}_{\mathrm{vir}}=0.80Q_{\max}$, $v_{5}=3.08\;\text{m}/\text{s},{\bar{Q}}_{\mathrm{vir}}=0.87Q_{\max}$, $v_{6}=3.19\;\text{m}/\text{s},{\bar{Q}}_{\mathrm{vir}}=0.90Q_{\mathrm{max.}}$

At exercise intensities that exceed the maximal aerobic power *Q*_*max*_, the virtual steady state is the steady state that would be attained if it was possible to carry out the exercise under purely aerobic conditions [27]. This virtual steady state is never reached as the aerobic power ends when *Q*_*max*_ is achieved. Figure 1 shows the steady state aerobic power ${\bar{Q}}_{a}$ for an elite skier running with poles at the angle of inclination of 0.105 radians on a large treadmill. A linear relationship applies for ${\bar{Q}}_{a}$ versus work rate up to approximately 95% of *Q*_*max*_. When repeating the experiment with the same skier, the difference did not exceed 25 W for this skier which is considered an upper bound error margin.

We defined a steady state virtual power ${\bar{Q}}_{\mathrm{vir}}$ which is a straight line that crosses *Q*_*max*_. Thus we set:

(3a) $${\bar{Q}}_{\mathrm{vir}}-Q_{r}\overset{model}{=}Q_{\mathrm{un}}+cP,{\bar{Q}}_{a}=Min\left(Q_{\max},{\bar{Q}}_{\mathrm{vir}}\right)\text{,}$$

(3b) $${\bar{P}}_{\mathrm{vir}}\overset{def}{=}\left({\bar{Q}}_{\mathrm{vir}}-Q_{r}\right)\eta_{a}\eta =\left(Q_{\mathrm{un}}+cP\right)\eta_{a}\eta ,\eta_{r}\overset{def}{=}1/\left(c\eta_{a}\eta \right)$$

where c is a parameter. *P* is the work rate. *Q*_*un*_ is the metabolic power due to unloaded running with poles. In general, *Q*_*un*_ depends on the angle of inclination and the cycle frequency of the activity. For a given incline the frequency depends of the work rate. Thus *Q*_*un*_ becomes a function of the work rate even for a fixed incline. ${\bar{P}}_{\mathrm{vir}}$ includes both internal and external work power. ${\bar{Q}}_{\mathrm{vir}}$ is input to the further modeling. We set that ${\bar{Q}}_{\mathrm{vir}}$ is a linear interpolation function through our data points. For values above or below the maximum and minimum measured ${\bar{Q}}_{\mathrm{vir}}$ we used linear extrapolation. During steady state, $Q_{a}^{w}={\bar{Q}}_{a}-Q_{r}=Min\left(Q_{\max}-Q_{r},{\bar{Q}}_{\mathrm{vir}}-Q_{r}\right)$.

The aerobic power is delayed with a time lag during steady state work rate. *Q*_*a*_(t) is the aerobic power and *Q*_*vir*_ is the virtual aerobic power, $Q_{a}\left(t\right)\overset{model}{=}Min\left(Q_{\max},Q_{\mathrm{vir}}\left(t\right)\right)$. To account for the delay mathematically, we used a first order differential equation of the virtual power, to read:

(4a) $${\overset{\cdot }{Q}}_{\mathrm{vir}}\left(t\right)\overset{model}{=}\frac{{\bar{Q}}_{\mathrm{vir}}-Q_{\mathrm{vir}}\left(t\right)}{\tau_{a}},\tau_{a}=30s,Q_{\mathrm{vir}}\left(t_{0}\right)=Q_{r}$$

(4b) $$Q_{a}\overset{model}{=}Min\left(Q_{\max},Q_{\mathrm{vir}}\left(t\right)\right)$$

The “dot” above a variable means time derivative. *τ*_*a*_ is the so-called *e*-folding time, which is the time interval when an exponentially growing quantity increases by a factor of *e*. Thus *τ*_*a*_ is a time parameter that scales from the initiation of the activity until the aerobic power reaches a steady state asymptotically when assuming a constant work rate. In practice, reaching steady state means having less than 1% change in aerobic power per second. The cardio dynamic phase is not necessary to account for since only the true aerobic power is used as input for our anaerobic models. Typical values for *τ*_*a*_ are shown to be 10-36 s for moderate intensity exercise [22,27-33]. For example, di Prampero [27] suggested 10-24 s, Ceretelli et al. [32] found that *τ*_*a*_ increases linearly with the concentration of lactate up to 36 s, and Bizoni et al. [33] found *τ*_*a*_ = 23 s for all work rates. We set that *τ*_*a*_ = 30 s as a compromise. Equation (4b) ensures that the aerobic power is less than the maximal aerobic power.

If the virtual metabolic power is equal to or below *Q*_*max*_ we have that $Q_{a}=Min\left(Q_{\max},Q_{\mathrm{vir}}\right)=Q_{\mathrm{vir}}$. This gives from equation (4a and b) that:

(5) $${\overset{\cdot }{Q}}_{a}\left(t\right)=\frac{{\bar{Q}}_{\mathrm{vir}}-Q_{a}\left(t\right)}{\tau_{a}}$$

For steady work rate equation (5) gives the analytical solution:

(6) $$Q_{a}\left(t\right)={\bar{Q}}_{\mathrm{vir}}-\left({\bar{Q}}_{\mathrm{vir}}-Q_{a}\left(t_{0}\right)\right)Exp\left(-\left(t-t_{0}\right)/\tau_{a}\right)$$

If the work rate is sufficiently low, ${\bar{Q}}_{a}={\bar{Q}}_{\mathrm{vir}}$. Notable is that solution (6) will only apply for a restricted time period unless ${\bar{Q}}_{a}={\bar{Q}}_{\mathrm{vir}}$.

The anaerobic power due to anaerobic glycolysis or glycogenolysis can be calculated from the total lactate concentration by using the equation from di Prampero and Ferretti [27], to read:

(7) $$Q_{G}\left(t\right)\overset{model}{=}m\times \lambda \times \overset{\cdot }{C}\left(t\right),\lambda =3\times 20:J/\left(kg\;mmol/L\right),\lambda =3ml/\mathrm{kg}\;O_{2},1mlO_{2}=20J$$

C is the lactate concentration defined as the amount of lactate per unit volume of lactate space (including muscles and blood). *m* is the mass of the body. λ converts the lactate per unit body mass to oxygen consumption. Note that during dynamic situations the blood lactate concentration differs significantly from the muscle lactate concentration. In this article, we measured the blood lactate concentration, which is different from the muscle lactate concentration. However, earlier research has demonstrated that the lactate concentration can be calculated as summarized in Appendix A [10].

The lactic energy used from time *t*_0_ to time *t* is:

(8) $$E_{G}\left(t_{0},t\right)\overset{def}{=}\int_{t_{0}}^{t}Q_{G}\left(t\prime \right)dt\prime =m\lambda \int_{t_{0}}^{t}\overset{\cdot }{C}\left(t\prime \right)dt\prime =m\lambda \left(C\left(t\right)-C\left(t_{0}\right)\right)$$

Experimentally, *E*_*G*_(*t*_0_, *t*) can be found by measuring changes in the lactate concentration before and after exercise.

From (2) we have that:

(9) Qaw+QG+QCK=1ηPa/ηa+PG/ηa+PCK/ηa+PG/ηG−PG/ηa+PCK/ηCK−PCK/ηa=PTηηa+PGη1/ηG−1/ηa︸QGt1−ηa/ηa+PCKη1/ηG−1/ηa︸QCKt1−ηCK/ηa⇒QCKt=ηaηCKPTtηηa−Qawt−ηGηaQGt

The metabolic power is then given by:

(10) $$\begin{array}{l} Q\left(t\right)-Q_{r}=Q_{a}^{w}+Q_{G}+Q_{\mathrm{CK}}=Q_{a}^{w}+Q_{G}+\frac{\eta_{a}}{\eta_{\mathrm{CK}}}\left(\frac{P_{T}}{\eta \eta_{a}}-Q_{a}^{w}-\frac{\eta_{G}}{\eta_{a}}Q_{G}\right)\\ \;=Q_{a}^{w}\left(t\right)\left(1-\frac{\eta_{a}}{\eta_{\mathrm{CK}}}\right)+Q_{G}\left(t\right)\left(1-\frac{\eta_{G}}{\eta_{\mathrm{CK}}}\right)+\frac{\eta_{a}}{\eta_{\mathrm{CK}}}\left(\frac{P_{T}\left(t\right)}{\eta \eta_{a}}\right) \end{array}$$

Steady state is achieved at exercise intensities below the lactate threshold. We defined steady state with the lactate concentration regarded as steady (i.e., no lactic power) and the aerobic power was steady, to read $\dot{C}=Q_{G}={\dot{Q}}_{a}^{w}=0$. Thus $Q_{a}^{w}$ is a constant and $Q-Q_{r}=Q_{a}^{w}$ during steady state. Hence, it follows that *Q*_*CK*_ = 0. Thus the alactic power is zero. To achieve *Q*_*CK*_ = 0 during steady state in (9) we must have $P_{T}=\eta \eta_{a}Q_{a}^{w}$. However, during steady state (below LT) $Q_{a}^{w}=Q_{\mathrm{un}}+cP$. Thus, below the lactate threshold $P_{T}=\left(Q_{\mathrm{un}}+cP\right)\eta \eta_{a}={\bar{P}}_{\mathrm{vir}}$ during steady state.

To close the equations in (9) and (10), which produce an indeterminate solution by themselves unless we have steady state, we needed a model for the work power *P*_*T*_ in general. As Model 1 we forecasted that the work power *P*_*T*_ was similar to the steady state virtual aerobic metabolic power. Thus, Model 1:

(11) $$P_{T}\left(t\right)\overset{model}{=}{\bar{P}}_{\mathrm{vir}}=\left({\bar{Q}}_{\mathrm{vir}}-Q_{r}\right)\eta_{a}\eta =\left(Q_{\mathrm{un}}+cP\right)\eta_{a}\eta$$

Inserting (11) into (9)-(10) gives the alactic power as:

(12a) $$\begin{array}{l} Q_{\mathrm{CK}}^{1}\left(t\right)=\frac{\eta_{a}}{\eta_{\mathrm{CK}}}\left({\bar{Q}}_{\mathrm{vir}}-Q_{r}-\left(Q_{a}\left(t\right)-Q_{r}\right)-\frac{\eta_{G}}{\eta_{a}}Q_{G}\left(t\right)\right)\\ \;=\frac{\eta_{a}}{\eta_{\mathrm{CK}}}\left({\bar{Q}}_{\mathrm{vir}}-Q_{a}\left(t\right)-\frac{\eta_{G}}{\eta_{a}}Q_{G}\left(t\right)\right) \end{array}$$

and the metabolic rate as:

(12b) $$Q^{1}\left(t\right)-Q_{r}=\left(Q_{a}\left(t\right)-Q_{r}\right)\left(1-\frac{\eta_{a}}{\eta_{\mathrm{CK}}}\right)+Q_{G}\left(t\right)\left(1-\frac{\eta_{G}}{\eta_{\mathrm{CK}}}\right)+\frac{\eta_{a}}{\eta_{\mathrm{CK}}}\left({\bar{Q}}_{\mathrm{vir}}-Q_{r}\right)$$

To calculate $Q_{\mathrm{CK}}^{1}\left(t\right)$ for a work rate *P*(t) in (12a) we inserted ${\bar{Q}}_{\mathrm{vir}}$ from (3), *Q*_*a*_(*t*) from (4) and *Q*_*G*_(*t*) from (7). It should be noted that the mechanical efficiency *η* is absent in (12a and b). We conceived that the work power *P*_*T*_ was proportional to the rate of consumption of ATP. Thus, an equivalent model to (11) would be that the rate of ATP consumption is linear with the work rate.

The alactic energy is for Model 1:

(13) $$\begin{array}{l} E_{\mathrm{CK}}^{1}(t_{0},t)\overset{def}{=}\int_{t_{0}}^{t}Q_{\mathrm{CK}}^{1}(t')dt'=\int_{t_{0}}^{t}\frac{\eta_{a}}{\eta_{\mathrm{CK}}}\left({\bar{Q}}_{\mathrm{vir}}-Q_{a}(t')-\frac{\eta_{G}}{\eta_{a}}Q_{G}(t')\right)dt'\\ \;=\frac{\eta_{a}^{}}{\eta_{\mathrm{CK}}}E_{\mathrm{DF}}(t_{0},t)-\frac{\eta_{G}}{\eta_{\mathrm{CK}}}E_{G}(t_{0},t)\\ E_{\mathrm{DF}}(t_{0},t)\overset{def}{=}\int_{t_{0}}^{t}\left({\bar{Q}}_{\mathrm{vir}}(t')-Q_{a}(t')\right)dt' \end{array}$$

The anerobic power and the anaerobic energy used from time $t_{0}$ to time t for Model 1 are:

(14a) $$\begin{array}{l} Q_{\mathrm{an}}^{1}(t)\overset{def}{=}Q_{\mathrm{CK}}^{1}(t)+Q_{G}(t)=\frac{\eta_{a}}{\eta_{\mathrm{CK}}}\left({\bar{Q}}_{\mathrm{vir}}-Q_{a}(t)-\frac{\eta_{G}}{\eta_{a}}Q_{G}(t)\right)+Q_{G}(t)\\ \;=\frac{\eta_{a}}{\eta_{\mathrm{CK}}}\left({\bar{Q}}_{\mathrm{vir}}-Q_{a}(t)\right)+\left(1-\frac{\eta_{G}}{\eta_{a}}\right)Q_{G}(t) \end{array}$$

and:

(14b) $$E_{\mathrm{an}}^{1}(t_{0},t)\overset{def}{=}\int_{t_{0}}^{t}Q_{\mathrm{an}}^{1}(t')dt'=\frac{\eta_{a}}{\eta_{\mathrm{CK}}}E_{\mathrm{DF}}(t_{0},t)+\left(1-\frac{\eta_{G}}{\eta_{a}}\right)E_{G}(t_{0},t)$$

where *E*_*DF*_(*t*_0_, *t*) is in the literature named the oxygen deficit of the exercise. Medbø et al. [18] and Losnegard et al. [29] calculated the anaerobic energy during exercise as *E*_*DF*_(*t*_0_, *t*) (assuming that the rate of change of O_2_ consumption is proportional to the aerobic power). Equation (14b) shows that $E_{\mathrm{DF}}\left(t_{0},t\right)=E_{\mathrm{an}}\left(t_{0},t\right)$ only if $\eta_{G}=\eta_{\mathrm{CK}}=\eta_{a}$. Thus, we deduced that the O_2_-deficit model for the anaerobic energy was equal to (14b) if the chemical coupling efficiencies were alike.

Model 1 is a general version of the O_2_-deficit model. As the O_2_-deficit model has been widely debated, we proposed an alternative in Model 2 where the lactic power and energy were the same as for Model 1. However, we hypothesized that the alactic power was proportional to the rate of change of the aerobic power [24,25]. Thus, for Model 2:

(15) $$Q_{\mathrm{CK}}^{2}(t)\overset{model}{=}\theta \;{\dot{Q}}_{a}(t)$$

where *θ* is a constant of proportionality parameter that we determine below. Equation (15) gives that:

(16) $$E_{\mathrm{CK}}^{2}\left(t_{0},t\right)\overset{def}{=}\int_{t_{0}}^{t}Q_{\mathrm{CK}}^{2}\left(t,\prime \right)dt\prime =\theta \int_{t_{0}}^{t}{\overset{\cdot }{Q}}_{a}\left(t\prime \right)dt\prime =\theta \left(Q_{a}\left(t\right)-Q_{a}\left(t_{0}\right)\right)$$

If the virtual metabolic power is below *Q*_*max*_ and ${\bar{Q}}_{a}={\bar{Q}}_{\mathrm{vir}}$, we achieved from equations (5) and (16) that:

(17) $$E_{\mathrm{CK}}^{2}\left(t_{0},t\right)=\theta \int_{t_{0}}^{t}{\overset{\cdot }{Q}}_{a}\left(t\prime \right)dt\prime =\frac{\theta }{\tau_{a}}\underset{Area}{\underset{︸}{\int_{t_{0}}^{t}\left({\bar{Q}}_{a}-Q_{a}\left(t\prime \right)\right)dt\prime }}=\theta \left(Q_{a}\left(t\right)-Q_{a}\left(t_{0}\right)\right)$$

The alactic energy can be found by calculating the area between ${\bar{Q}}_{a}$ and *Q*_*a*_ in a power time diagram. This area is multiplied with *θ*/*τ*_*a*_ to find the alactic energy used. *θ*/*τ*_*a*_ can be considered as the effectiveness of alactic ATP production relative to the aerobic ATP production. Thus we set $\theta /\tau_{a}=\eta_{a}/\eta_{\mathrm{CK}}$.

The mechanical efficiency was assumed to be *η* ≈ 0.5. For the chemical efficiency related to aerobic or lactic power $\eta_{G}^{}=\eta_{a}^{}\text{are}\text{between}0:3\text{and}0:7$. We used $\eta_{G}=\eta_{a}\approx 0.6$. This gave $\eta \times \eta_{G}=\eta \times \eta_{a}=0.3$. Since *c* was around 6.6 we found that $\eta_{r}=1/\left(c\times \eta \times \eta_{G}\right)=1/\left(6.6\times 0.5\times 0.6\right)=0.51$. Gonzales-Alonso et al. [26] concluded from experimental data that the heat per use of ATP was around two times larger for oxidative phosphorylation and anaerobic glycolysis compared to ATP from CK. For the alactic power we therefore forecasted that *η*_*CK*_ = 0.95. This gave that $\theta =\left(\eta_{a}/\eta_{\mathrm{CK}}\right)\tau_{a}=20\;s$.

It has been shown that during intensities above the lactate threshold, ATP utilization increases and mechanical efficiency decreases at constant work rates. This may be explained by a change in fiber type recruitment, an elevated temperature, lowered pH or increased Pi levels [30]. We forecasted that *τ*_*a*_, *η*, *η*_*a*_, *η*_*G*_ and *η*_*CK*_ mainly depend on the lactate concentration and change in muscle pH, and assumed that a lower mechanical efficiency of muscle contractions and a lower P/O_2_ ratio could explain the drift in the steady state ATP consumption for a given work rate. To account for time varying parameters see the model in Appendix B.

Increased fitness after exercise is developed during the recovery period after the exercise. We defined an exercise which starts at time *t*_0_ and ends at time *t*_1_, and a subsequent recovery period which starts at time *t*_1_ and ends at time *t*_2_. Time *t*_2_ is defined as $Q_{a}\left(t_{2}\right)=Q_{a}\left(t_{0}\right)$ and *C*(*t*_0_) = *C*(*t*_2_). This means that $E_{G}\left(t_{0},t_{2}\right)=0$ and $E_{G}\left(t_{0},t_{1}\right)=-E_{G}\left(t_{1},t_{2}\right)$. We defined −*E*_*DF*_(*t*_1_, *t*_2_) as the energy depth *E*_*DB*_(*t*_1_, *t*_2_), to read:

(18) $$\begin{array}{l} E_{\mathrm{DB}}\left(t_{1},t_{2}\right)\overset{def}{=}-E_{\mathrm{DF}}\left(t_{1},t_{2}\right)=-\int_{t_{1}}^{t_{2}}\left(Q_{a}\left(t\prime \right)-{\bar{Q}}_{\mathrm{vir}}\right)dt\prime \text{,}\\ \;EPOC\left(t_{1},t_{2}\right)\overset{def}{=}-\int_{t_{1}}^{t_{2}}\left(Q_{a}\left(t\prime \right)-{\bar{Q}}_{a}\right)dt\prime \end{array}$$

We also defined the so-called excess post–exercise oxygen consumption (EPOC). In general *E*_*DB*_(*t*_1_, *t*_2_) is different from *EPOC*(*t*_1_, *t*_2_) unless ${\bar{Q}}_{\mathrm{vir}}={\bar{Q}}_{a}$. However, the latter is usually the case during recovery.

For Model 1 in equations (13) we achieved:

(19) $$\begin{array}{l} E_{\mathrm{CK}}^{1}\left(t_{0},t_{2}\right)=\frac{\eta_{a}}{\eta_{\mathrm{CK}}}E_{\mathrm{DF}}\left(t_{0},t_{2}\right)-\frac{\eta_{G}}{\eta_{\mathrm{CK}}}\underset{=0}{\underset{︸}{E_{G}\left(t_{0},t_{2}\right)}}\\ =\frac{\eta_{a}}{\eta_{\mathrm{CK}}}E_{\mathrm{DF}}\left(t_{0},t_{1}\right)+\frac{\eta_{a}}{\eta_{\mathrm{CK}}}E_{\mathrm{DF}}\left(t_{1},t_{2}\right)=0\Rightarrow E_{\mathrm{DF}}\left(t_{0},t_{1}\right)=-E_{\mathrm{DF}}\left(t_{1},t_{2}\right)=E_{\mathrm{DB}}\left(t_{1},t_{2}\right) \end{array}$$

In this case the alactic energy used from time *t*_0_ to time *t*_1_ for Model 1 was therefore given by:

(20) $$E_{\mathrm{CK}}^{1}\left(t_{0},t_{1}\right)=\frac{\eta_{a}}{\eta_{\mathrm{CK}}}E_{\mathrm{DB}}\left(t_{1},t_{2}\right)+\frac{\eta_{G}}{\eta_{\mathrm{CK}}}E_{G}\left(t_{1},t_{2}\right)=\frac{\theta }{\tau_{a}}E_{\mathrm{DB}}\left(t_{1},t_{2}\right)+\frac{\eta_{G}}{\eta_{\mathrm{CK}}}E_{G}\left(t_{1},t_{2}\right)$$

For Model 2 we have:

(21) $$\begin{array}{l} E_{\mathrm{CK}}^{2}\left(t_{0},t_{2}\right)=\int_{t_{0}}^{t_{2}}Q_{\mathrm{CK}}^{2}\left(t\prime \right)dt\prime =\theta \int_{t_{0}}^{t_{2}}{\overset{\cdot }{Q}}_{a}\left(t\prime \right)dt\prime \\ \;=\underset{E_{\mathrm{CK}}^{2}\left(t_{0},t_{1}\right)}{\underset{︸}{\theta \int_{t_{0}}^{t_{1}}{\overset{\cdot }{Q}}_{a}\left(t\prime \right)dt\prime }}+\underset{E_{\mathrm{CK}}^{2}\left(t_{1},t_{2}\right)}{\underset{︸}{\theta \int_{t_{1}}^{t_{2}}{\overset{\cdot }{Q}}_{a}\left(t\prime \right)dt\prime }}=0\Rightarrow E_{\mathrm{CK}}^{2}\left(t_{0},t_{1}\right)=-E_{\mathrm{CK}}^{2}\left(t_{1},t_{2}\right) \end{array}$$

We defined *EPOC*_*Alt*_ as an alternative variant of the excess post-exercise oxygen consumption:

(22) $$EPOC_{\mathrm{Alt}}\left(t_{1},t_{2}\right)\overset{def}{=}-\tau_{a}\int_{t_{1}}^{t_{2}}{\overset{\cdot }{Q}}_{a}\left(t\prime \right)dt\prime =\tau_{a}\left(Q_{a}\left(t_{1}\right)-Q_{a}\left(t_{2}\right)\right)$$

Thus, finally:

(23) $$\begin{array}{l} E_{\mathrm{CK}}^{2}\left(t_{0},t_{1}\right)=\int_{t_{0}}^{t_{1}}Q_{\mathrm{CK}}^{2}\left(t\prime \right)dt\prime =\theta \int_{t_{0}}^{t_{1}}{\overset{\cdot }{Q}}_{a}\left(t\prime \right)dt\prime =\frac{\theta }{\tau_{a}}\times \tau_{a}\left(Q_{a}\left(t_{1}\right)-Q_{a}\left(t_{2}\right)\right)\\ \;=\frac{\theta }{\tau_{a}}\times EPOC_{\mathrm{Alt}}\left(t_{1},t_{2}\right) \end{array}$$

Here, the alactic energy used from time *t*_0_ to time *t*_1_, could be found by measuring the aerobic power at time *t*_1_ and *t*_2_. The respective aerobic powers could be subtracted and then multiplied with *θ* to find the alactic energy.

It was also of interest to compare Model 1 and Model 2 more directly when *Q*_*vir*_ = *Q*_*a*_ and ${\bar{Q}}_{\mathrm{vir}}={\bar{Q}}_{a}$ . When using equation (5) this gave:

(24) $$\begin{array}{l} If\;Q_{\mathrm{vir}}=Q_{a}and\;{\bar{Q}}_{\mathrm{vir}}={\bar{Q}}_{a},\text{then }EPOC_{\mathrm{Alt}}\left(t_{1},t_{2}\right)=-\tau_{a}\int_{t_{1}}^{t_{2}}{\overset{\cdot }{Q}}_{a}\left(t\prime \right)dt\prime \\ =-\int_{t_{1}}^{t_{2}}\left(Q_{a}\left(t\prime \right)-{\bar{Q}}_{\mathrm{vir}}\right)dt\prime =E_{\mathrm{DB}}\left(t_{1},t_{2}\right)=-\int_{t_{1}}^{t_{2}}\left(Q_{a}\left(t\prime \right)-{\bar{Q}}_{a}\right)dt\prime =EPOC\left(t_{1},t_{2}\right) \end{array}$$

From equations (20)-(23) this gave:

(25a) $$\begin{array}{l} E_{\mathrm{CK}}^{1}\left(t_{0},t_{1}\right)=\frac{\eta_{a}}{\eta_{\mathrm{CK}}}E_{\mathrm{DB}}\left(t_{0},t_{1}\right)+\frac{\eta_{G}}{\eta_{\mathrm{CK}}}E_{G}\left(t_{1},t_{2}\right)\\ \;=\underset{first\;term}{\underset{︸}{\frac{\theta }{\tau_{a}}EPOC\left(t_{1},t_{2}\right)}}+\underset{second\;term}{\underset{︸}{\frac{\eta_{G}}{\eta_{\mathrm{CK}}}E_{G}\left(t_{1},t_{2}\right)}} \end{array}$$

(25b) $$E_{\mathrm{CK}}^{2}\left(t_{0},t_{1}\right)=\frac{\theta }{\tau_{a}}EPOC\left(t_{1},t_{2}\right)$$

These two methods gave different results due to fact that the second term for $E_{\mathrm{CK}}^{1}\left(t_{0},t_{1}\right)$ lacks in Model 2.

Hill et al. [34-37] have explained that the oxidative removal of lactate increases aerobic power. Using equation (25a) we achieved that:

(26) $$\begin{array}{l} E_{\mathrm{an}}^{1}\left(t_{0},t_{1}\right)=\int_{t_{0}}^{t_{1}}Q_{\mathrm{an}}^{1}\left(t\prime \right)dt\prime =E_{\mathrm{CK}}^{1}\left(t_{0},t_{1}\right)+E_{G}^{1}\left(t_{0},t_{1}\right)\\ =\frac{\eta_{a}}{\eta_{\mathrm{CK}}}E_{\mathrm{DF}}\left(t_{0},t_{1}\right)+\left(1-\frac{\eta_{G}}{\eta_{a}}\right)E_{G}\left(t_{0},t_{1}\right)=\frac{\theta }{\tau_{a}}EPOC\left(t_{1},t_{2}\right)+\left(\frac{\eta_{G}}{\eta_{a}}-1\right)E_{G}\left(t_{1},t_{2}\right) \end{array}$$

If *η*_*G*_ = *η*_*a*_, *EPOC*(*t*_1_, *t*_2_) corresponds to the total anaerobic energy, i.e. lactic and alactic anaerobic energy. Indeed, Margaria et al. [38] later on modified the concept of Hill et al. [34-37], and suggested that the increased aerobic power consisted of the fast alactic component and the slower lactic component. Finally, Gaesser and Brooks [39] introduced the term “excess post-exercise oxygen consumption”, which also included the much more prolonged increase in aerobic power that is observed for hours after exercise. Model 1 did not account for this very slow component that lasts for hours. Model 2 in equation (25b) said that EPOC corresponded to the alactic component. However, the development of the energy in equation (25b) depended of the time dynamics for the aerobic power modeled according to equation (4).

### Experimental tests

The derived mathematical simulations were compared with experimental data from an elite cross-country skier while running with poles on a treadmill. The mass of the skier was *m* = 77.5 kg in all tests. All treadmill tests were performed on a 6 × 3 m motor-driven treadmill (Bonte Technology, Zwolle, The Netherlands). Inclination and speed were calibrated using the Qualisys Pro Reflex system and the Qualisys Track Manager software (Qualisys AB, Gothenburg, Sweden). The treadmill belt consisted of a non-slip rubber surface that allowed the skier to use his own poles (pole length: 80% of body height) with special carbide tips. Gas exchange values were measured by open-circuit indirect calorimetry using an Oxycon Pro apparatus with a mixing chamber (Jaeger GmbH, Hoechberg, Germany). Before each measurement, the VO_2_ and VCO_2_ gas analyzers were calibrated using high-precision gases (16.00 ± 0.04% O_2_ and 5.00 ± 0.1% CO_2_, Riessner-Gase GmbH & co, Lichtenfels, Germany). The inspiratory flow meter was calibrated with a 3 L volume syringe (Hans Rudolph Inc., Kansas City, MO). Heart rate (HR) was measured with a heart rate monitor (Polar S610, Polar Electro OY, Kempele, Finland), using a 5 s interval for data storage. Blood lactate concentration (BLa) was measured on 5 μL samples taken from the fingertip by a Lactate Pro LT-1710 *t* (ArkRay Inc, Kyoto, Japan).

Eight experimental protocols were performed, each on separate days with a minimum of 48 h between. The order of tests was performed as presented below. In order to investigate whole body exercise, running with poles was used in all tests. Before each testing session a standardized, test-specific 20-min warm-up was performed. Training on the days before testing was standardized, and the subject drank a standard fluid with sugar and electrolytes during all breaks while testing.

On the first test day, the skier performed six 5 min bouts with constant work rates at 0.105 inclines in radians. Five speeds at 0.25 m/s intervals below the lactate threshold were chosen, starting at 7.5 km/h = 2.08 m/s, followed by 2.33 m/s, 2.58 m/s, 2.83 m/s and 3.08 m/s. The sixth speed was increased by 0.125 m/s, giving 3.19 m/s, which is slightly above the lactate threshold. The starting speed was chosen based on experience from earlier tests of this athlete. 5-min bouts were used to obtain steady state conditions. A 2-min break with low-intensity walking was mandatory between each of the exercise bouts. Gas exchange values and heart rates were determined by the average of the last minute during each stage and blood lactate was measured directly after finishing each stage. The reason for using the last minute to assess respiratory variables was that the athletes are able to keep a more steady technique and physiological responses after 3-4 min.

In follow-up tests, we aimed to investigate whether our simulations fitted experimental data when varying the exercise time and during recovery after high-intensity exercise. Also, velocities slightly above or below the lactate threshold were tested in order to test the sensitivity of our model for lactate. Thus, the skier performed the following six tests on separate days with at least 2 days in between each test, at 0.105 radians incline with blood lactate measured directly after each stage:

– 2.87 m/s was performed for 800 s.

– 3.08 m/s was performed over 800 s, followed by a 5-min recovery phase at 1.67 m/s with blood lactate measured after 2 and 5 min. Additionally, a 3.05 m/s stage was performed to exhaustion.

– 3.08 m/s was performed over 2000 s.

– 3.19 m/s stages were performed for 400 s and to exhaustion.

– A 3.33 m/s stage was performed for 400 s.

– 3.88 m/s stages were performed for 150 s, 200 s and to exhaustion.

Finally, maximal metabolic power was tested on a separate day at an incline of 0.105 radians with a starting speed of 3 m/s. The speed was increased by 0.3 m/s every minute until exhaustion. VO_2_ was measured continuously, and the average of the three highest 10 s consecutive measurements determined VO_2_max and was used to calculate the maximal metabolic power. The test was considered to be a maximal effort if the following three criteria were met: 1) a plateau in VO_2_ was obtained with increasing exercise intensity, 2) respiratory exchange ratio above 1.10, and 3) blood lactate concentration exceeding 8 mmol/L.

## Results

**Figure 2:**
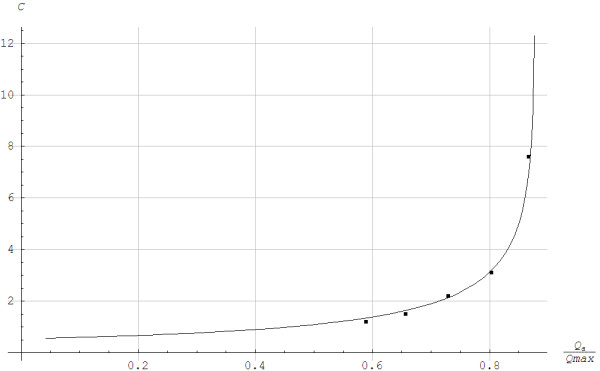
**Steady state lactate concentration *C *in mmol/L as a function of the fraction of the maximum aerobic power *Q***_***max***_**when running with poles at the angle of inclination of 0.105 for 5-min steady state work rates.**$v_{1}=2.08\;\text{m}/\text{s},{\bar{Q}}_{\mathrm{vir}}=0.59\;Q_{\max}$, $v_{2}=2.33\;\text{m}/\text{s},{\bar{Q}}_{\mathrm{vir}}=0.66\;Q_{\max}$, $v_{3}=2.64\;\text{m}/\text{s},{\bar{Q}}_{\mathrm{vir}}=0.74\;Q_{\max}$, $v_{4}=2.83\;\text{m}/\text{s},{\bar{Q}}_{\mathrm{vir}}=0.80\;Q_{\max}$, $v_{5}=3.08\;\text{m}/\text{s},{\bar{Q}}_{\mathrm{vir}}=0.87\;Q_{\max}$, $C=C_{s}=\frac{1}{2}Ln\left(\frac{1+\frac{p_{0}\chi }{d_{0}Q_{\max}\left(1-Q_{a}/Q_{\max}\right)}}{1-\frac{p_{0}\chi }{d_{0}Q_{\max}\left(1-Q_{a}/Q_{\max}\right)}}\right)/\chi$, $\chi =2.54\;m^{3}/\mathrm{kg},p_{0}=10^{-5}kg/\left(m^{3}s\right)/\left(J/s\right),d_{0}=1.1\;10^{-7}/{\left(J/s\right)}^{2}/s,Q_{\max}=1886J/s,Q_{\mathrm{LT}}/Q_{\max}=0.88$.

Figure 2 illustrates that the experimental steady state blood lactate values showed good similarity with the simulated values based on Moxnes and Hausken’s [10] one compartment model for the average concentration in the total lactate pool. The blood lactate concentration equals the average concentration during steady state. At high lactate concentrations, the drain of lactate is saturated, which can be modeled by the function $Tanh\left(\chi \;C\left(t\right)\right)$. No saturation was achieved when *χ*→0, since $\underset{\chi \to 0}{Lim}\;Tanh\left(\chi \;C\left(t\right)\right)/\chi =C\left(t\right)$. The lactate threshold *Q*_*LT*_ was achieved when $1-\frac{\chi p_{0}}{d_{0}\left(Q_{\max}-Q_{a}\right)}=0\Rightarrow Q_{a}=Q_{\mathrm{LT}}=Q_{\max}-\frac{\chi p_{0}}{d_{0}},\frac{Q_{\mathrm{LT}}}{Q_{\max}}=1-\frac{\chi p_{0}}{d_{0}Q_{\max}}$. *Q*_*max*_ was known and we used the numerical value of *p*_0_ from Moxnes and Hausken [10]. Thus, we only needed to fit the two parameters *χ*/*d*_0_ and *χ* to the steady state measured values.

In the first step, we applied visual curve fitting, which means that we chose plausible values for the two parameters. We then plotted and compared the solution visually with the experimental data. The values of the two parameters were changed repeatedly until a good visual fit was obtained, while ensuring that the parameters had physiologically trustworthy numerical values. In the second step, a least square fit to the data was performed to produce ex post best fit estimates of these two parameters using the visual estimates as starting guess points and choosing a range around each starting point of the parameters. The method was performed separately for each parameter keeping the other parameter fixed. Steps 1 and 2 were repeated sufficiently until we were certain that we had obtained the optimal values for each of the two parameters. For robustness in the calculations, we applied the steady state solution. If we applied that $C_{s}\approx 1+\frac{p_{0}}{d_{0}Q_{\max}\left(1-Q_{a}/Q_{\max}\right)}$, the parameter *χ* got removed. The lactate threshold became *Q*_*a*_ = *Q*_*max*_. *χ* determined the lactate threshold by the exact relation. The rest of the lactate curve was dependent on $\frac{p_{0}}{d_{0}Q_{\max}\left(1-Q_{a}/Q_{\max}\right)}$. This gave a linear dependency of the lactate curve on *p*_0_/*d*_0_ and an inverse dependency of the lactate curve on *Q*_*max*_ − *Q*_*a*_.

**Figure 3:**
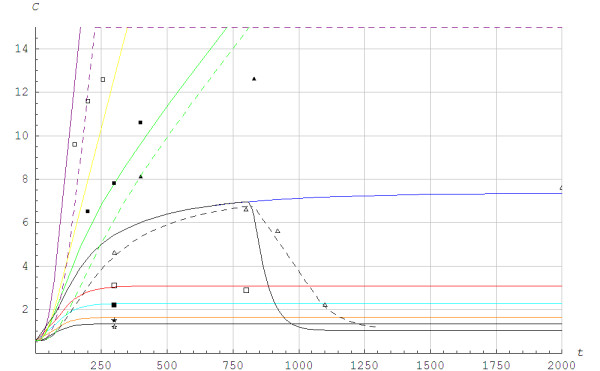
**The theoretical and experimental blood lactate concentration *C *in mmol/L as a function of time *t *in seconds when running with poles at the angle of inclination of 0.105 radians.** The eight symbols from below are experimental values from the different tests v_1-8_. The straight curves represent results from a one-compartment model by Moxnes and Hausken [2], whereas the three stapled curves represent results from the two compartment model by Moxnes and Sandbakk [25] for v_5_, v_6_ and v_8_. Lines from below: *black =*$v_{1}=2.08\;\text{m}/\text{s},{\bar{Q}}_{\mathrm{vir}}=0.59\;Q_{\max}$, *orange =*$v_{2}=2.33\;\text{m}/\text{s},{\bar{Q}}_{\mathrm{vir}}=0.66\;Q_{\max}$, *cyan =*$v_{3}=2.64\;\text{m}/\text{s},{\bar{Q}}_{\mathrm{vir}}=0.74\;Q_{\max}$, *red =*$v_{4}=2.83\;\text{m}/\text{s},{\bar{Q}}_{\mathrm{vir}}=0.80\;Q_{\max}$, *blue =*$v_{5}=3.08\;\text{m}/\text{s},{\bar{Q}}_{\mathrm{vir}}=0.87\;Q_{\max}$ until a recovery phase starts at *t* = 800s with *v* = 1.67m/s, ${\bar{Q}}_{\mathrm{vir}}=0.47Q_{\max}$, *green =*$v_{6}=3.19\;\text{m}/\text{s},{\bar{Q}}_{\mathrm{vir}}=0.90\;Q_{\max}$, *yellow =*$v_{7}=3.33\;\text{m}/\text{s},{\bar{Q}}_{\mathrm{vir}}=0.94\;Q_{\max}$, *pink =*$v_{8}=3.88\;\text{m}/\text{s},{\bar{Q}}_{\mathrm{vir}}=1.09\;Q_{\max}$*.*$\text{LT}=0.88\;Q_{\mathrm{max.}}$

Figure 3 demonstrates the measured and simulated lactate concentration during steady state work rate. In this simulation, we only applied visual fitting to determine the *d*_0_ parameter, and found that the simulated lactate concentration was somewhat higher than the measured (blood lactate) concentration for intensities above the lactate threshold. During recovery, the measured blood lactate concentration was higher than the simulated lactate concentration. Thus, we also calculated values according to Moxnes and Sandbakk [22] where the lactate concentration was separated into two compartments: muscles + internal organs and blood. In that case, Figure 3 shows that the simulation results are more similar to the experimental results. However, some discrepancies still appear for the higher work rates.

**Figure 4:**
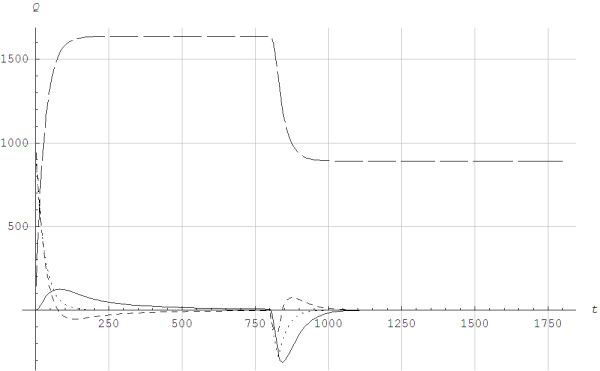
**Powers *Q *in J/s as function of time *t *in seconds when running with poles at the angle of inclination of 0.105 radians.***v* = 3.08 m/s, ${\bar{Q}}_{\mathrm{vir}}=0.87Q_{\max}$ when *t* < 800 s. *v* = 1.67 m/s, ${\bar{Q}}_{\mathrm{vir}}=0.47\;Q_{\max}$ when *t* > 800 s. ____ ___: Aerobic power $\left(Q_{a}=Q_{b}+Q_{a}^{w}\right)$, ________: Lactic anaerobic power (*Q*_*G*_), -----------: Alactic anaerobic power (*Q*_*CK*_) for Model 1, .........: Alactic anaerobic power (*Q*_*CK*_) for Model 2.

Figure 4 shows the different anaerobic alactic powers for intensities below the lactate threshold when using Models 1 and 2. The calculated alactic power for Model 1 showed a somewhat complicated behavior since it also depended on the model for the lactic power. A special feature was the overshoot that applied during recovery because the lactic power becomes negative. For Model 2 the lactic power was only different from zero when the rate of change of the aerobic power was different from zero.

**Figure 5:**
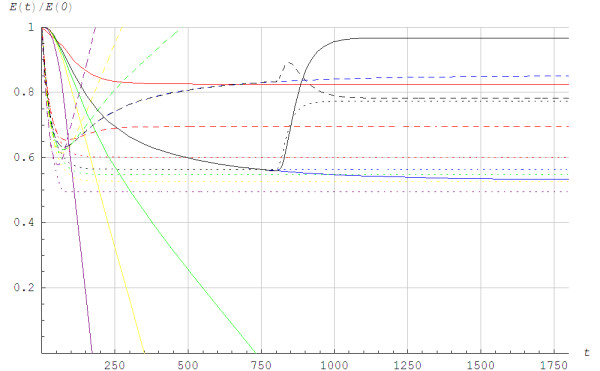
**The lactic and alactic anerobic energy utilization *E*(*t*)/*E*(0) as a function of time *t *in seconds for different work rates when running with poles at the angle of inclination of 0.105.** ______: Lactic energy utilization $E_{G}\left(t\right)/E_{G}\left(0\right),E_{G}\left(0\right)=870J/kg\times m$, - - - - -: Alactic energy utilization $E_{\mathrm{CK}}\left(t\right)/E_{\mathrm{CK}}\left(0\right),E_{\mathrm{CK}}\left(0\right)=830J/kg\times m$ using Model 1, ......: Alactic energy utilization $E_{\mathrm{CK}}\left(t\right)/E_{\mathrm{CK}}\left(0\right),E_{\mathrm{CK}}\left(0\right)=830J/kg\times m$ using Model 2. From below in different colors: *pink =*$v_{8}=3.88\;\text{m}/\text{s},{\bar{Q}}_{\mathrm{vir}}=1.09\;Q_{\max}$*yellow =*$v_{7}=3.33\;\text{m}/\text{s},{\bar{Q}}_{\mathrm{vir}}=0.94\;Q_{\max}$, *green =*$v_{6}=3.19\;\text{m}/\text{s},{\bar{Q}}_{\mathrm{vir}}=0.90\;Q_{\max}$, *blue =*$v_{5}=3.08\;\text{m}/\text{s},{\bar{Q}}_{\mathrm{vir}}=0.87\;Q_{\max}$, *black =*$v_{5}=3.08\;\text{m}/\text{s},{\bar{Q}}_{\mathrm{vir}}=0.87\;Q_{\max}$ until a recovery phase starts at *t* = 800s with *v* = 1.67m/s, ${\bar{Q}}_{\mathrm{vir}}=0.47Q_{\max}$, *red =*$v_{4}=2.83\;\text{m}/\text{s},{\bar{Q}}_{\mathrm{vir}}=0.80\;Q_{\max}$, The exercises are terminated for *v*_8_, *v*_7_ and *v*_6_.

Figure 5 illustrates the lactic and alactic storage of energy. We found different behaviors for Models 1 and 2. For Model 1, the alactic energy storage started to increase from a local minimum at the time the aerobic power reached a steady state. The aerobic power reached steady state before the lactic power did for *v*_4_ and *v*_5_. When the exercise was terminated for *v*_6_, *v*_7_ and *v*_8_, the alactic energy was fully restored. The alactic energy resources increased due to use of lactic power. Although the details of the time history of alactic energy depend on the chemical coupling coefficients, the overall scenario was much the same for different numerical values of the coefficients. It is important to note that the alactic power depended on the model for the lactic power. When O_2_ was excluded, the PCr store was rebuilt by anaerobic glycogenolysis or glycolysis [15-17]. In Model 2, the alactic energy showed a simpler behavior than in Model 1 since the alactic power only depended on the rate of change of the aerobic power. When comparing the solution in Figure 5 in the current paper with the invasive measurements shown in Figure 2 in the paper Jeneson et al. [23] it appeared that Model 2 gave a better fit than Model 1.

To study the kinetics of anaerobic powers more in detail we compared interval exercise with continuous exercise by using Models 1 and 2. These were simulated exercises matched for energy expended. Interval exercise changed in intensity with durations above and below the lactate threshold as described below. The continuous exercise maintained steady state intensity. The two exercises were given by:

$$\begin{array}{l} Interval:{\bar{Q}}_{\mathrm{vir}}=Q_{\max}/2+\left(Q_{\max}/2\right)\times Sin\left(2\pi t/T\right)\text{,}\;T=360s\\ Continuous:{\bar{Q}}_{\mathrm{vir}}=\left\{ \begin{array}{l} Q_{\max}/2+\left(Q_{5}-Q_{\max}/2\right)\times Sin\left(2\pi t/T\right)\;when\;0\leq t\leq 1/4T\\ Q_{\max}/2+\left(Q_{5}-Q_{\max}/2\right)\;when\;1/4T<t\leq T \end{array}\right.\\ \;Q5=0.87\;Q_{\max} \end{array}$$

**Figure 6:**
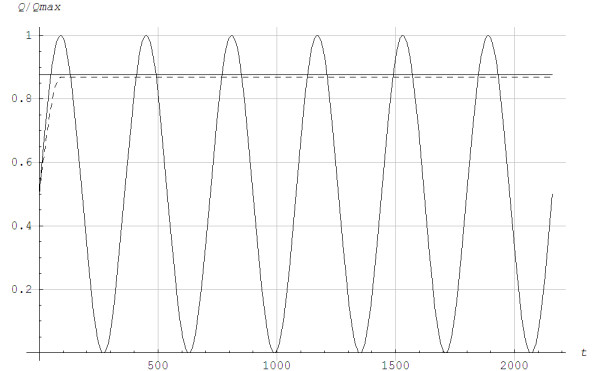
**The metabolic power as a fraction of the maximum aerobic power *Q/Qmax *as a function of time *t *in seconds for an interval and a continuous exercise.** The horizontal lines represent the lactate threshold. -------: Continuous. _____: Interval.

**Figure 7:**
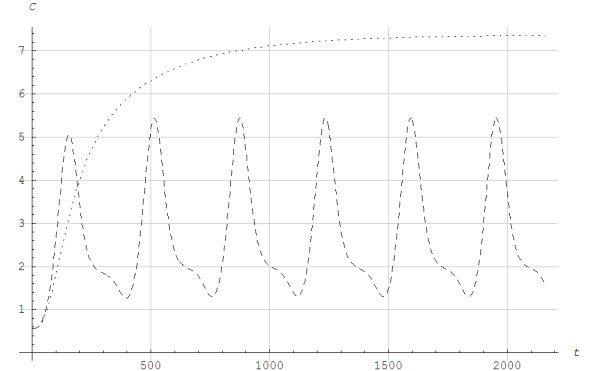
**The lactate concentration *C *in mmol/L as a function of time *t *in seconds for interval and continuous exercise during the protocol showed in Figure**6. ....: Continuous. -------: Interval.

**Figure 8:**
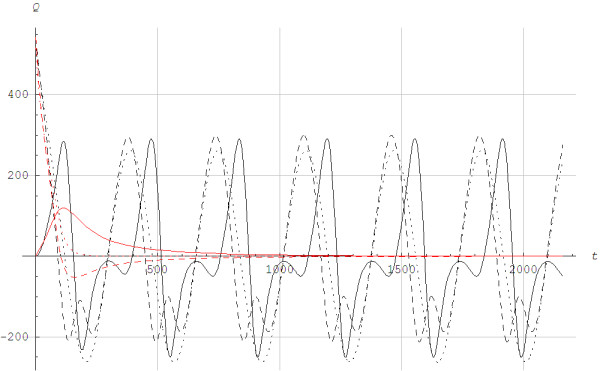
**The anaerobic lactic and alactic powers (*Q*) in J/s as a function of time *t *in seconds for interval (black lines) and continuous (red lines) exercise during the protocol showed in Figure**6. ______: Lactic anaerobic power (*Q*_*G*_). ---------: Alactic anaerobic power (*Q*_*CK*_), Model 1. ......: Alactic anaerobic power (*Q*_*CK*_), Model 2.

**Figure 9:**
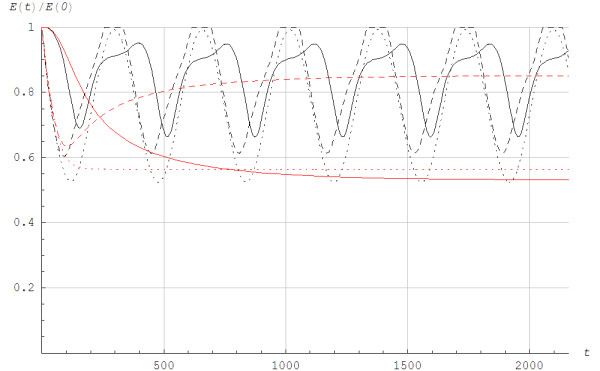
**Calculations of the energy utilization *E*(*t*)/*E*(0) as a function of time *t *in seconds for (black lines) and continuous (red lines) exercises during the protocol showed in Figure**6. _______: Lactic anaerobic energy utilization $E_{G}\left(t\right)/E_{G}\left(0\right),E_{G}(0)=870J/kg\times m$. - - - - - -: Alactic anaerobic energy utilization $E_{\mathrm{CK}}\left(t\right)/E_{\mathrm{CK}}\left(0\right),E_{\mathrm{CK}}\left(0\right)=830J/kg\times m$ Model 1. ......: Alactic anaerobic energy utilization $E_{\mathrm{CK}}\left(t\right)/E_{\mathrm{CK}}\left(0\right),E_{\mathrm{CK}}\left(0\right)=830J/kg\times m$ Model 2.

*T* is the time variable, 0<=t<=T, and T = 360 s is a parameter for the time duration, i.e. how long the exercise lasted. Figure 6 shows the metabolic powers for interval and continuous exercises as functions of time, whereas Figure 7 shows the simulated lactate concentration for the two different exercises. The lactate values were reduced to the initial levels between intervals, whereas lactate continued to rise during continuous exercise. Figure 8 shows the lactic power and the alactic powers calculated by Models 1 and 2. Overall, the alactic energy stores were rebuilt during recovery with interval exercise, which allowed for the alactic energy to be used at the beginning of each interval. Figure 9 shows the lactic and alactic energy utilization and shows that during exercise the alactic energy storage decreased with time for Model 2. For Model 1 a local minimum appeared around the time when the aerobic power reached steady state. During interval exercise, the alactic energy storage as a function of time did not differ much between the two models.

## Conclusions

The current study investigated two different mathematical models for the kinetics of anaerobic power during whole body exercise at different exercise intensities. The results indicate that blood lactate levels can be accurately modeled during steady state, and suggest a linear relationship between the alactic anaerobic power and the rate of change of the aerobic power. Overall, we propose that the current simulation models provide useful insight into how the anaerobic powers during practical training and testing should be interpreted.

When comparing the experimental blood lactate values with Moxnes and Hausken’s [10] model for lactic power, we found similar results during steady state exercise intensities. Uncertainties of less than ± 0.5 mmol/L using these mathematical models are indicated. For intensities above the lactate threshold, the measured blood lactate values were significantly lower than those calculated by the Moxnes and Hausken [10] model, whereas during recovery after high intensity exercise, the blood lactate concentration was higher than calculated. This could be explained by a possible delay in blood lactate concentration compared to the average concentration in the total lactate pool that applies theoretically. Therefore, we compared the experimental results with a two-compartment model by Moxnes and Sandbakk [22], where lactate concentration was separated into muscle and blood compartments. The employment of this model gave a better fit between simulated and experimental results, especially for the highest work rates and during recovery. However, some discrepancies still appeared for the higher work rates, which is a topic for further research.

The calculation of alactic storage of energy in Model 1, which is a generalization of the O_2_-deficit model, showed that the alactic energy storage increased due to use of lactic power from the time the aerobic power reached a steady state. However, the various inferences about the alactic storage of energy during steady state work rate have some shortcomings. For example, the results may have been influenced by the fact that the O_2_-deficit is composed of both lactic and alactic metabolisms. Thus, no definite conclusion could be drawn for Model 1 for alactic power. For Model 2, the alactic energy showed a simpler behavior since it only depended on the rate of change of the aerobic power. When comparing Models 1 and 2 with the invasive measurements by Jeneson et al. [23], we concluded that Model 2 gave the best behavior. Thus, a linear relationship between the alactic anaerobic power and the rate of change of the aerobic power was suggested. In future research, it remains a challenge to confirm the contribution of lactic and alactic anaerobic metabolism and power.

The simulations of interval exercises showed a drop in lactate levels between the intervals, and that the alactic energy stores were rebuilt during recovery. Practically, this allows athletes to work at high exercise intensities for longer time durations without lowering the pH values in the muscles compared to continuous exercise at similar intensities. This might be one reason for the effectiveness of interval exercise, as verified both by the scientific literature [40] and by elite athletes’ practical training programs where interval exercises are an important component. When the alactic anaerobic powers during continuous exercises from Models 1 and 2 were compared, Model 1 showed a local minimum appearing around the time when the aerobic power reached steady state for continuous exercise, whereas for Model 2 the alactic energy storage decreased with time. During interval exercise, the alactic energy storage as a function of time was similar for the two models.

Modeling in human biology is a challenge since one is confronted with conceiving a simple, but realistic representation of complex phenomena. The parameter values used in the current study will be dependent on the fitness level of the individual being tested and on the concentration of glycogen in the muscles. To construct valid simulation models during dynamic exercise such models need to be developed for each individual.

### Appendix A: The lactate model

In equation (25) of Moxnes and Hausken [10] we find that:

(A1) $$\begin{array}{l} \overset{\cdot }{C}\left(t\right)=p_{0}Q_{a}\left(t\right)\;p_{1}\left(Q_{a}\left(t\right)\right)\\ \;-d_{0}Q_{a}\left(t\right)\left(Q_{\max}-Q_{a}\left(t\right)\right)d_{1}\left(Q_{a}\left(t\right)\right)\frac{\left(1-Exp\left(-\alpha \;C\left(t\right)\right)\right)}{\alpha } \end{array}$$

where *p*_1_() and *d*_1_() are dimensionless functions and *α* is a parameter to describe the saturation of the concentration of lactate *C*(*t*). We use $\frac{Tanh\left(\chi \;C\left(t\right)\right)}{\chi }$ instead of $\frac{\left(1-Exp\left(-\alpha \;C\left(t\right)\right)\right)}{\alpha }$ to describe saturation. To read:

(A2) $$\begin{array}{l} \overset{\cdot }{C}\left(t\right)=p_{0}D\left(Q_{a}\left(t\right)\right)-d_{0}\times \frac{Tanh\left(\chi \;C\left(t\right)\right)}{\chi }\times D\left(Q_{a}\left(t\right)\right)\times \left(Q_{\max}-Q_{a}\left(t\right)\right)\\ D\left(Q_{a}\left(t\right)\right)\overset{\text{def}}{=}Q_{a}\left(t\right)\;p_{1}\left(Q_{a}\left(t\right)\right)=d_{1}\left(Q_{a}\left(t\right)\right) \end{array}$$

We set that:

(A3) $$D\left(Q_{a}\right)\overset{mod}{=}Q_{a}-\frac{\alpha_{0}}{\beta_{0}p_{0}}Tanh\left(\beta_{0}p_{0}Q_{a}\right)$$

and *β*_0_ is a parameter that must be fitted to the experimental data, as earlier done by Moxnes and Sandbakk [22] when studying a two compartment model of lactate. The steady state solution is given by:

(A4) $$C_{s}=\frac{1}{2\chi }Ln\left(\frac{1+\frac{p_{0}\chi }{d_{0}Q_{\max}\left(1-Q_{a}/Q_{\max}\right)}}{1-\frac{p_{0}\chi }{d_{0}Q_{\max}\left(1-Q_{a}/Q_{\max}\right)}}\right)\text{,}\;\underset{\chi \to 0}{Lim}C_{s}=\frac{p_{0}}{d_{0}Q_{\max}\left(1-Q_{a}/Q_{\max}\right)}$$

We use $1/\left(\beta_{0}\;p_{0}\right)=0.6\;Q_{\max}$, where 0.6*Q*_*max*_ is around peak fat metabolism. All together we set:

(A5) $$\begin{array}{l} D\left(x\right)=\left(x-\alpha_{0}0.6\;Q_{\max}Tanh\left(\frac{x}{0.6\;Q_{\max}}\right)\right),1/\left(\beta_{0}p_{0}\right)=0.6\;Q_{\max},\alpha_{0}=0.9,\\ C\left(t_{0}\right)=0.045kg/m^{3}=0.5\;m\;mol/L,\\ \chi =2.54\;m^{3}/kg,p_{0}=10^{-5}kg/(m^{3}s)/(J/s),d_{0}=1.1\times 10^{-7}/{\left(J/s\right)}^{2}/s,Q_{\max}=1886J/s \end{array}$$

### Appendix B: Model for parameters varying with the lactate level

To account for situations with varying *τ*_*a*_, *η*_*a*_, *η*_*G*_ and *η*_*CK*_ we can use a first order differential equation of the virtual aerobic metabolic power, to read:

(B1) $$\begin{array}{l} {\overset{\cdot }{P}}_{\mathrm{vir}}\left(t\right)\overset{\text{model}mod}{=}\frac{{\bar{P}}_{\mathrm{vir}}-P_{\mathrm{vir}}\left(t\right)}{\tau_{a}},P_{a}\overset{\text{model}}{=}Min\left(\eta \eta_{a}\left(Q_{\max}-Q_{b}\right),P_{\mathrm{vir}}\left(t\right)\right),\tau_{a}=30s,P_{\mathrm{vir}}\left(t_{0}\right)=0,\\ Q_{a}=P_{a}/\left(\eta \eta_{a}\right) \end{array}$$

Equation (B1) allows the calculation of the aerobic power when the efficiencies and the time parameter *τ*_*a*_ are time dependent. This might appear due to changes in lactate levels. We assume that *η* × *η*_*a*_ is constant through time. This gives from (B1):

(B2) $$\begin{array}{l} {\overset{\cdot }{P}}_{\mathrm{vir}}\left(t\right)/\left(\eta \eta_{a}\right)=\frac{\left({\bar{P}}_{\mathrm{vir}}/\left(\eta \eta_{a}\right)-P_{\mathrm{vir}}\left(t\right)/\left(\eta \eta_{a}\right)\right)}{\tau_{a}}\\ \;\Rightarrow {\overset{\cdot }{Q}}_{\mathrm{vir}}\left(t\right)=\frac{\left(\overset{{\bar{Q}}_{\mathrm{vir}}}{\overset{︷}{{\bar{P}}_{\mathrm{vir}}/\left(\eta \eta_{a}\right)}}-Q_{\mathrm{vir}}\left(t\right)\right)}{\tau_{a}}=\frac{\left({\bar{Q}}_{\mathrm{vir}}-Q_{\mathrm{vir}}\left(t\right)\right)}{\tau_{a}},\\ Q_{a}\overset{\text{model}}{=}Min\left(Q_{\max},Q_{\mathrm{vir}}\left(t\right)\right),\left(b\right),\tau_{a}=30s,Q_{\mathrm{vir}}\left(t_{0}\right)=Q_{b} \end{array}$$

## Competing interests

The authors declare that they have no competing interests.

## Authors’ contributions

JM performed the mathematical simulations, ØS performed all laboratory testing and KH supported in finishing the paper in its final form. All authors contributed with important intellectual content in all parts of the manuscript. All authors read and approved the final manuscript.
